# Antibacterial potential of essential oils of *Zataria multiflora* and *Mentha piperita*, micro- and nano-formulated forms

**Published:** 2020-02

**Authors:** Mahmoud Osanloo, Abbas Abdollahi, Alireza Valizadeh, Niloufar Abedinpour

**Affiliations:** 1 Department of Medical Nanotechnology, School of Advanced Technologies in Medicine, Fasa University of Medical Sciences, Fasa, Iran; 2 Department of Microbiology, School of Medicine, Fasa University of Medical Sciences, Fasa, Iran; 3 Department of Medical Nanotechnology, School of Advanced Technologies in Medicine, Tehran University of Medical Sciences, Tehran, Iran; 4 Department of Medicine, School of Medicine, Fasa University of Medical Sciences, Fasa, Iran

**Keywords:** *Zataria multiflora*, *Mentha piperita*, Essential oil, Antibacterial activity, Nanoemulsion

## Abstract

**Background and Objectives:**

Plant-derived essential oils (EOs) shave many usages in health and medicine, such as antibacterial agents. The aim of this study was the improvement of antibacterial activities of two EOs using nanotechnology.

**Materials and Methods:**

Antibacterial activity was investigated on four important human pathogenic bacteria using the 96-well plate microdilution method, a quantitative approach. Eleven formulations were prepared using each of the EOs. Eventually, the best nanoformulation with the smallest particle size and polydispersive indices (PDI and SPAN) was selected using each EO for further investigations. Moreover, two microemulsions with similar ingredients and the same portion in comparison with two selected nanoemulsions were also prepared. Antibacterial activity of each EO was compared with its micro- and nano-emulsions.

**Results:**

The antibacterial efficacy of Zataria multiflora EO (ZMEO) was significantly better than Mentha piperita EO (MPEO). Besides, the antibacterial activity of nanoemulsion of ZMEO with a particle size of 129 ± 12 nm was significantly better than no- and micro-formulated forms of ZMEO. Interestingly, the efficiency of MPEO nanoemulsion (160 ± 25 nm) was also significantly better than MPEO and its micro-formulated form.

**Conclusion:**

Regardless of the intrinsic antibacterial property of two examined EOs, by formulating to nanoemulsion, their efficiencies were improved. Nanoemulsion of ZMEO introduced as an inexpensive, potent and green antibacterial agent.

## INTRODUCTION

Nanotechnology defined as targeted manipulations of materials in nanoscale for obtaining size-dependent features or functions (1). The most common nanomaterials are metallic nanoparticles (2), polymeric nanoparticles (3), lipidic nanocarriers (4) and nanoemulsions (5). Nanoemulsions are submicron-sized emulsions (generally 1–200 nm) in which two immiscible liquid (aquatic and oil phases) are mixed to form a single-phase, using one or more surfactants with or without using external energy such as ultrasound or probe homogenizer (6). The repeatable and straightforward manners for preparation of nanoemulsions with small and monodisperse particles has led to the widespread use of those in agriculture (7), health (8) and medicine (9).

Essential oils (EO)s are natural aromatic compounds derived from different parts of plants such as bark and stem (10). EO shave been widely used in health and medicine for many years, e.g., for larvicidal activity (11), antifungal purpose (12), antiparasitic research (13) and antibacterial effect (14).

In this research, antibacterial activities of two medicinally important plant-derived EOs, including *Zataria multiflora* (ZMEO) and *Mentha piperita* (MPEO), were investigated using the microdilution method. Targeted bacteria were some of the important pathogens, i.e., *Staphylococcus aureus, Escherichia coli, Pseudomonas aeruginosa* and *Klebsiella pneumoniae*. Moreover, we tried to improve the antibacterial activities of the EOs by formulating them into the nanoemulsions dosage form. Also, by preparing the microemulsion of ZMEO and MPEO, examined the effect of particle size on the antibacterial effect.

## MATERIAlS AND METHODS

Standard species of bacteria, including *S. aureus* (ATCC 25923), *E. coli* (ATCC 25922), *P. aeruginosa* (ATCC 27853), and *K. pneumoniae* (ATCC 13883) were supplied by the laboratory of microbiology, Fasa University of Medical Sciences (FUMS). ZMEO and MPEO were bought from Zardband pharmaceutical Co, Iran.

**The procedure of GC-MS analysis.** The EOs components were identified using GC-MS analysis, which described in our previous report (5).

**Investigation of antibacterial activity of EOs.** 96-well plate microdilution method was used for determining the growth inhibitory effect of EOs against target bacteria with slight modification (15). Briefly, new bacterial colonies were dissolved in a defined amount of nutrient broth (2×; Concentration twice as standard) to reach 0.5 McFarland (1.5 × 10^8^ CFU/mL) turbidity in 630 nm by 0.08 to 0.1 optical density. Then 20 and 80 μL of the bacterial suspension and the nutrient broth, respectively, were added to each well of the plate using an 8-channel pipette.

A stock solution of each EO was prepared by dissolving in normal-saline (NS) at a concentration of 4000 μg.mL^−1^ (noted, at a higher level, EOs did not dissolve in NS). Then, serial dilutions of ZMEO and MPEO were prepared with a two-dimensional dilution of stock solutionin NS for developing concentration ranges of 4000-62.5 μg.mL^−1^. By the addition of 100 μL from serial dilutions to each well, the concentration of EOs finally fixed at 2000, 1000, 500, 250, 125, 62.5 and 31.25 μg.mL^−1^. Plates were then incubated at 37°C for 24 hours, and then absorption of wells was read at 630 nm using a plate reader (Synergy HTX-Multi-Mode Reader, USA). The tests were repeated three times, and in each replicate, six wells considered as control and blank groups. For the control groups, 20, 80 and 100 μL form the bacteria suspension, the nutrient broth, and NS respectively, was added to each well. Blank wells contained nutrient broth and NS (100: 100 μL). Using the following equation, growth (%) of bacteria at each concentration was determined.

Growth (%) = A sample - A blank / A Control - A blank × 100

*A: Mean absorption

**Fig. 1.**
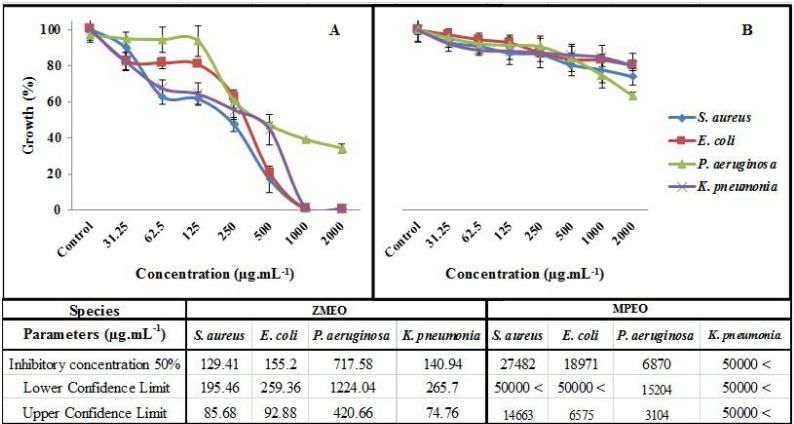
Bacteria growth inhibitory activity of ZMEO (A) and MPEO (B) and related factors

**Preparation of EOs nanoemulsions.** Many components of EOs are volatile. Thus, the spontaneous method was used for preparing nanoemulsions (16). A defined amount of ZMEO or MPEO (separately) and tween 20 were entirely mixed at room temperature to form a homogenous solution (500 rpm, 10 min). NS was then added dropwise to the mixture up to the desired volume (i.e., 5000 μL). The prepared mixture was stirred at 1500 rpm for 30 min. For the preparation of emulsions, eleven amounts of tween 20 (as a surfactant) and NS (as aqueous phase) were-used (0–50 and 4940–4995 μL, respectively). The highest concentration of each EO, which its growth inhibitory activity on all targeted bacteria was close together, was chosen for the preparation of emulsion. These amounts for ZMEO and MPEO included 250 and 500 μg.mL^−1^ (Fig. 1A and B).

Because the emulsions should be diluted during the antibacterial test, the formulations were made using 4× more EOs. Used amounts of ZMEO and MPEO in their formulations (with the volume of 5000 μL) were fixed at 5 and 10 μL respectively. In other words, by addition of 50 μL of such emulsion into each well containing 150 μL other substrates (i.e., bacteria, NS and nutrient broth), the concentration of ZMEO and MPEO fixed at the mentioned concentrations (250 and 500 μg.mL^−1^, respectively).

**Characterization of prepared emulsions: analyses of size.** The mean diameter of particle sizes (PS), polydispersity index (PDI) and particle size distributions (SPAN) of prepared emulsions were determined using nanoparticle size analyzer apparatus (SZ-100 series, HORIBA Scientific, Japan). For each of EO, nanoformulation with lowest PS, PDI and SPAN was selected as optimum nanoemulsion for antibacterial tests.

**Investigation of size effect on antibacterial activity.** Another study was designed to investigate the impact of emulsion size on antibacterial activity. For this purpose, two microemulsions with similar components (with the same portion) to optimized nanoemulsions with bigger particle sizes (PS, PDI and SPAN) were also prepared. In the preparation procedure of those emulsions, were named microemulsion, NS was added at one-shot instead of dropwise addition.

**Comparison of antibacterial activity of EOs with their nano/micro-emulsions.** Comparisons of antibacterial activities of ZMEO and MPEO with their nano/micro-emulsion were investigated at 250 and 500 μg.mL^−1^, respectively, as detailed in previous sections with slight modifications. In the first step, 50 μL from each EO and it's micro- and nano-emulsion was added to wells, separately. Then 80, 50 and 20 μL of nutrient broth (2×), NS and the prepared bacteria suspension were added to each well, respectively. Then plates were incubated at 37°C for 24 hours and absorption was read at 630 nm using a plate reader. Using equation 1, the bacterial growth inhibitory activity of each sample was calculated.

## RESUlTS

**Components of EOs.** Thirty-eight components were identified in ZMEO using GC-MS analysis with five major components including carvacrol (30.23%), thymol (25.20%), o-cymene (10.73%), gamma-terpinene (6.13%) and alpha.-pinene (3.61%) (Data not given). However, among the 52 identified components in MPEO, menthol, L-menthone, camphane, menthofuran and Iso-menthone had a more substantial portion (31.08, 22.11, 7.03, 6.02 and 5.86%, respectively) in comparison with others (Data not given).

**Bacteria growth inhibitory activity of EOs.** Bacterial growth inhibitory effects of ZMEO on targeted strains are demonstrated in Fig. 1(A). Bacteria were exposed 24 hours with a concentration range of each EO, separately (i.e., 31.25–2000 μg.mL^−1^). IC50 of ZMEO against *S. aureus, E. coli, P. aeruginosa* and *K. pneumoniae* were observed at 129.41, 155.2, 717.58 and 140.94 μg.mL^−1^, respectively. Lower and upper confidence limits of mentioned IC50 are also given in Fig. 1. Interestingly, at concentrations of 1000 and 2000 μg.mL^−1^ growth of *S. aureus, E. coli* and *K. pneumoniae* reduced to ∼ 0%. Furthermore, the antibacterial properties of MPEO are also shown in Fig. 1(B). The inhibitory effect of MPEO (IC50) against *S. aureus, E. coli, P. aeruginosa* and *K. pneumoniae* was achieved as 27482, 18971, 6870 and 5000 < μg.mL^−1^, respectively. For other factors, including lower and upper confidence limits, see Fig. 1.

**Prepared emulsions and selecting optimum nanoemulsions.** Details of 22 prepared ZMEO and MPEO emulsions are given in Table 1. All emulsions were prepared at defined volume (5000 μL) using different amounts of tween 20 and NS (as an aqueous phase). Noted that, due to the lower antibacterial activity of MPEO in comparison with ZMEO, the used amount of that was fixed at 10 μL (instead of 5 μL).

**Table 1.** Prepared MPEO and MPEO emulsions: ingredients and characteristics

**Formulation**	**Formulation ingredients**				**Prepared formulations characterization**		

	**MPEO (μl)**	**ZMEO (μl)**	**Tween 20 (μl)**	**NS (μl)**	**PS (nm)**	**PDI**	**SPAN**
MP1	10	0	0	4990	MPEO was not dispersed		
MP2	10	0	5	4985	MPEO was not dispersed		
MP3	10	0	10	4980	147 ± 23	0.361 ± 0.04	2.068 ± 2.06
MP4	10	0	15	4975	180 ± 18	0.347 ± 0.01	1.775 ± 0.23
MP5	10	0	20	4970	160 ± 25	0.495 ± 0.07	0.714 ± 0.26
MP6	10	0	25	4965	14 ± 14	0.939 ± 0.16	0.931 ± 0.22
MP7	10	0	30	4960	83 ± 18	4.516 ± 2.6	49.942 ± 7.12
MP8	10	0	35	4955	2142 ± 394	3.578 ± 0.33	2.876 ± 2.87
MP9	10	0	40	4950	129 ± 18	0.894 ± 0.13	1.680 ± 1.33
MP10	10	0	45	4945	10 ± 1.5	1.045 ± 0.83	15.312 ± 2.13
MP11	10	0	50	4940	19 ± 20	4.517 ± 0.99	0.742 ± 0.56
ZM1	0	5	0	4995	ZMEO was not dispersed		
ZM2	0	5	2.5	4992.5	12 ± 1	4.91 ± 062	0.13 ± 0.03
ZM3	0	5	5	4990	129 ± 12	0.15 ± 0.11	0.68 ± 0.27
ZM4	0	5	7.5	4987.5	4551 ± 155	12.84 ± 0.47	0.41 ± 0.43
ZM5	0	5	10	4985	160 ± 30	0.34 ± 0.26	0.82 ± 0.64
ZM6	0	5	12.5	4982.5	369 ± 53	30.83 ± 42.67	1.19 ± 0.24
ZM7	0	5	15	4980	1 ± 1	14.61 ± 1.54	0.16 ± 0.05
ZM8	0	5	17.5	4977.5	6 ± 3	10.91 ± 4.07	0.18 ± 0.02
ZM9	0	5	20	4975	6 ± 6	7.94 ± 5.49	0.15 ± 0.04
ZM10	0	5	22.5	4972.5	10 ± 3	6.59 ± 4.67	0.11 ± 0.01
ZM11	0	5	25	4970	2 ± 0	8.45 ± 3.71	0.66 ± 0.92

**Fig. 2.**
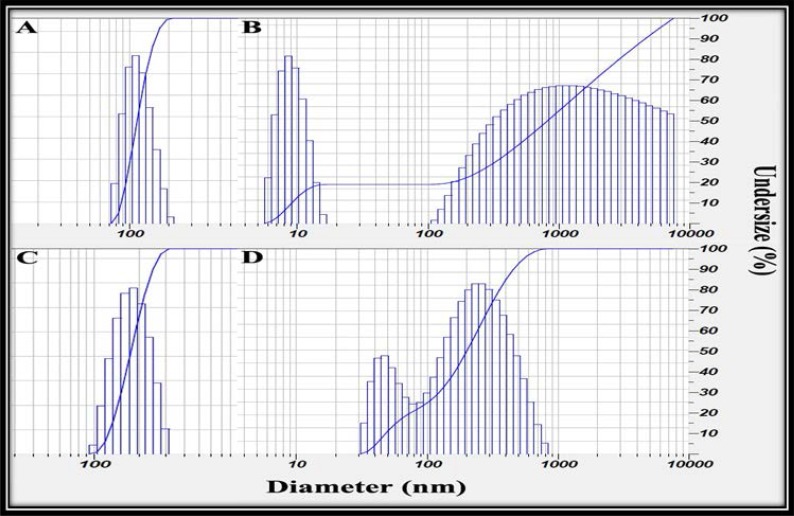
Size analyses of selected emulsions (PS, PDI and SPAN): A: ZM3NF (129 ± 12, 0.15 ± 0.11 and 0.68 ± 0.27), B: ZM3MF (1580 ± 42, 4.530 ± 0.9 and 5.58 ± 1) C: MP5NF (160 ± 25, 0.495 ± 0.07 and 0.714 ± 0.26) and MP5MF (230 ± 28, 0.349 ± 0.5 and 2.04 ± 0.8).

To select the optimum nanoformulation, PS should be less than 200 nm, and distribution factors must be within acceptable limits, i.e., PDI < 0.7 and SPAN < 1. In this regard, one formulation form each EO was selected as optimum nanoemulsions. They called ZM3 NF, and MP5 NF and their DLS analyses are illustrated in Fig. 2 (A and C), respectively.

**Comparison of size of the prepared micro- and nano-emulsions.** For evaluating size effect on antibacterial activity, microemulsions (with the same ingredients and similar amounts) with bigger PS, PDI and/or SPAN in comparison to the selected nanoemulsions were also prepared. They called ZM3 MF and MP5 MF (see Fig. 2 (B and D)). PS of ZM3 MF and MP5 MF were 580 ± 42 and 230 ± 28 nm, respectively. Other size parameters of ZM3 MF include PDI and SPAN, were 4.530 ± 0.9 and 5.58 ± 1.0, respectively. Furthermore, the PDI value for MP5 MF was 0.349 ± 0.2, and SPAN was 2.043 ± 0.81.

**Fig. 3.**
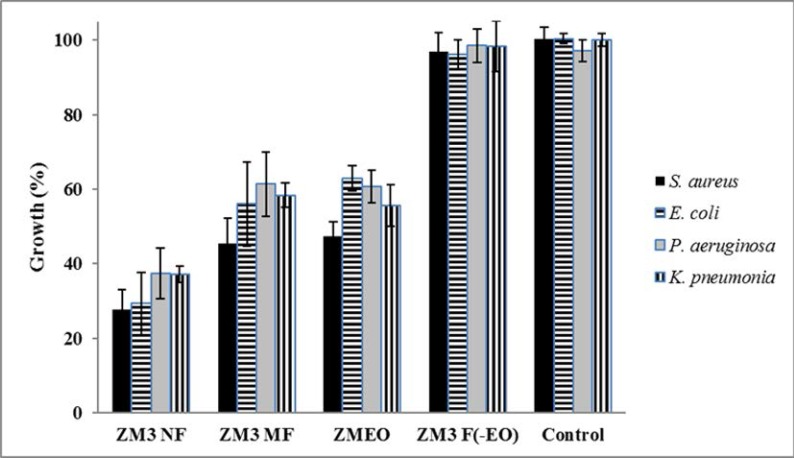
Comparison of antibacterial activities of ZMEO in comparison its nano-and micro-emulsions and ingredients of emulsions (ZM3NF, ZM3MF and ZM3 F(-EO)) at 250 μg.mL^−1^

**Comparison of bacterial growth inhibitory activity of each EO in comparison with its micro- and nano-emulsions.** Fig. 3. compares the antibacterial activity of ZMEO with its micro- and nano-emulsions at a concentration of 250 μg.mL^−1^. As details show, the growth of all bacteria exposed to ZM3 NF was significantly lower than ZM3 MF and ZMEO (one-way ANOVA, *p* < 0.05). However, no significant difference was seen between ZMEO and ZM3 MF (independent sample t-test, *p* > 0.05). Additionally, no significant difference (independent sample t-test, *p* > 0.05) was viewed between the ingredient of micro/nano-emulsion (ZM3 F(-EO)) and control group, which implied, had no significant impact on the growth of bacteria.

**Fig. 4.**
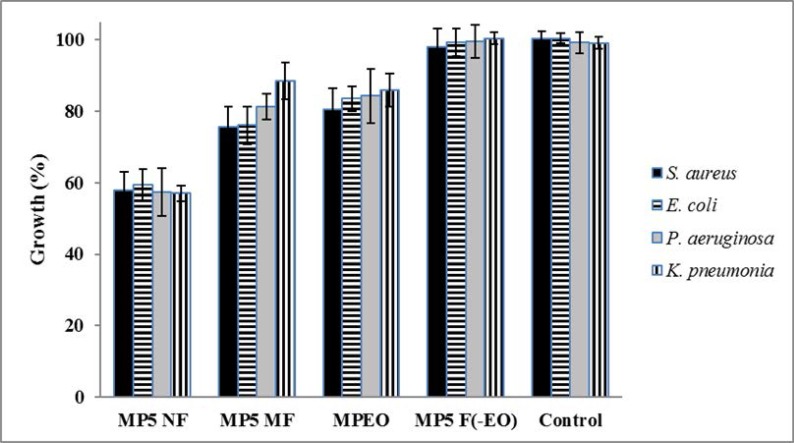
Comparison of antibacterial activities of MPEO in comparison with its nano-and micro-emulsions and emulsions ingredients (MP5 NF, MP5 MF and MP5 F(-oil)) at 500 μg.mL^−1^

From Fig 4, the inhibitory effect of MP5 NF was significantly higher than its correspond microemulsion (MP5 MF) and non-formulated EO (MPEO) (one-way ANOVA, *p* < 0.05). Also, ingredients used for the preparation of nano/micro-emulsion had no significant impact on the growth of bacteria in comparison with the control group (independent sample t-test, *p* > 0.05).

## DISCUSSION

Comparing obtained IC50s of ZMEO and MPEO on target bacteria, the antibacterial activity of ZMEO was significantly better than MPEO on all examined bacteria (Independent sample t-test, *p* < 0.05). Due to the high efficiency of ZMEO to control at least three-important types of bacteria, it introduced as a potent antibacterial substrate.

Investigating antibacterial activities of ZMEO and MPEO using qualities manner was performed previously. For example, minimum inhibitory concentration (MIC) of them against *E. coli* was reported as 42 and 1.25 mg.mL^−1^, respectively, while this value for *S. aureus* was 21 and 2.5 mg.mL^−1^ respectively (17, 18). Reviewing the literature demonstrated that IC50 of ZMEO was at lower concentration compared to *Citrus reticulate* (540 ± 10 μg.mL^−1^), *Citrus grandis* (1100 ± 50 μg.mL^−1^), and *Cinnamomum zeylanicum* (2350 ± 90 μg.mL^−1^) against *E. coli* (19). However, the effectiveness of some of the other EOs was better than ZMEO. For instance, IC50 of *Ferula ovina, Ferula akitsckensis* and *Ferula iliensis* on *S. aureus* were reported as 19.1 ± 2.9, 47.8 ± 4.7 and 94.3 ± 11.1 μg.mL^−1^, respectively (20).

From the literature, some papers have been found on investigating the antibacterial activity of major components of MPEO and ZMEO. For instance, MIC of menthol (major parts of MPEO) against *S. aureus* were reported as 0.62 and 0.63 mg.mL^−1^ and against *E. coli* was 2.50 and 1.25 mg.mL^−1^ (21). Moreover, the antibacterial activities of thymol and carvacrol have been reported frequently, e.g., their MIC on *Streptococcus salivarius* was observed at 5 and 2.5 mg.mL^−1^, respectively (22). Furthermore, the MIC of thymol on *S. aureus* and *E. coli* were reported as 0.31 and 5.00 mg.mL^−1^, respectively (21). The fact that the main components of MPEO and ZMEO have antibacterial properties is a reasonable justification for confirming the antibacterial properties of the EOs. However, due to differences in the method of evaluation of antibacterial activity in the mentioned studies and this study, the effect of the main components on the antibacterial activity of the two essential oils needs further investigation.

As details are shown in Table 1, without using tween 20, ZMEO was not dispersed homogeneously in NS, even with 2 hours’ exposure with ultrasound (Data not shown). Tween 20 at a higher amount of 12.5 μL in 5000 μL had a significant impact on increasing PDI; emulsions (ZM7–11) with very small PS (i.e., 1–10 nm) but with not acceptable PDI (6–14). Moreover, like to ZMEO emulsions, by the increasing amount of tween 20 in MPEO formulations (MP9–11), one or more of factors (i.e., PDI or SPAN) had out of acceptable values. Implied that micelle droplets (without EO) in emulsions were formed (16).

For obtaining optimum nanoformulation having lower and acceptable PS, PDI and PSD, balancing between components are necessary (23). Among the prepared emulsions of ZMEO, just ZM3 and ZM5 meet the mentioned conditions. Finally, ZM3 (PS (129 ± 12), PDI (0.15 ± 0.11) and SPAN (0.68 ± 0.27)) was selected as optimum nanoemulsion due to a significant lowering of PDI in comparison with ZM5 (0.15 ± 0.11 < 0.64 ± 0.26) and using a lower amount of tween 20: 5 μL instead of 10 μL (lowering its cost). Among the prepared formulations of ADEO, MP5 with the smallest amounts of PS, PDI and SPAN (160 ± 25 nm, 0.495 ± 0.07 and 0.714 ± 0.26, respectively) was selected as optimum MPEO nanoemulsion.

Nowadays, it is accepted that the encapsulation of EO at the nanoscale (1–200 nm) leads to enhancing the physical stability of bioactive compounds and increasing their bioactivity (24, 25). However, another possible mechanism for improvingthe performance of nanoemulsion compared with EO or microformulations is related to better dispersion of EO droplets in the water phase (26). So, higher contact between bacterial cells and EO droplets is expected at alower size (15). Furthermore, in this study, nanoemulsion and microemulsion were made using similar components with the same portion. Thus, the only difference between them was size. It seems small PS and better monodispersity of the nanoemulsions helped them to penetrate the bacterial cells better and damage the bacterial cell wall (in comparison with micro- and non-formulated EO) (27).

Similar results with the present study can be found, i.e., better performance of nanoemulsion in comparison with non-formulated EO. For instance, MIC (%) of nanoemulsion of *Lemon myrtle* significantly lower than EO on *S. aureus* (0.062 and 0.156), *Listeria monocytogenes* (0.031 and 0.156), and *E. coli* (0.25 and 0.625) (24). Antibacterial activity (MIC%) of nano emulsion (NE) of clove EO on targeted bacteria, including *Bacillus subtilis, Proteus vulgaris, S. aureus, P. aeruginosa* and *K. pneumoniae* were reported as 0.080, 0.085, 0.075, 0.300 and 0.250, respectively, while MIC of EO was significantly higher, i.e., 0.130, 0.130, 0.130, 0.500 and 0.400, respectively (28). Antimicrobial activity of NE and non-formulated EO of *Cymbopogon flexuosus* against different microorganism were reported as *Candida albicans* (0.28 and 1.22 mg/mL), *Cryptococcus grubii* (0.28 and 0.58 mg/mL), *P. aeruginosa* (11.33 mg/mL and Not active), and *S. aureus* (0.58 and 0.58 mg/mL) (29).

## CONClUSION

In this research it was confirmed that by formulating two examined EO into nanoemulsion, their antibacterial activity has improved. Furthermore, the antibacterial activity of nanoemulsion is significantly better than its microemulsion (with the same ingredients and amounts). Moreover, the nanoemulsion of *Zataria multiflora* EO is introduced as a new antibacterial substrate due to its proper potency and green components.
